# Comparison between automated and manual digital diagnostic setups of orthodontic extraction cases: an in silico study

**DOI:** 10.1186/s40510-026-00605-6

**Published:** 2026-02-02

**Authors:** Taghrid K. Barbary, Walid A. Elkenany, Yomna M. Yacout

**Affiliations:** Alexandria University, Alexandria, Egypt

**Keywords:** Virtual setup, Computer simulation, Computer-assisted diagnosis, Artificial intelligence, Digital dental model

## Abstract

**Background:**

The aim of the study was to evaluate automated digital diagnostic setup in bimaxillary dentoalveolar protrusion cases using two software packages and to compare them to manual digital setup.

**Methodology:**

Pre-treatment intraoral scans of 14 patients whose treatment plans involved extraction of four first premolars were imported as Standard Tessellation Language files into dentOne^®^ software (DIORCO co. ltd, Yongin, South Korea) and Ortho Simulation software (MEDIT Corp, Seoul, South Korea). Following tooth segmentation and selection of the teeth to be extracted, an automatic virtual setup was performed in each software. Moreover, manual virtual setups were performed by an orthodontist using dentOne^®^ software. Dental arch changes and dental movements and the duration taken to perform the setups were evaluated and compared using the appropriate statistical tests.

**Results:**

The inter-canine, inter-premolar and inter-molar widths did not change significantly following manual virtual setup, while the arch length significantly decreased. The inter-premolar width, inter-molar width and arch length significantly decreased following both automated setups. The manual setup showed significantly greater lingual translation of maxillary and mandibular incisors compared to Ortho Simulation software (mean difference = 5.97 ± 1.10 mm and 7.02 ± 1.29 mm, respectively) and dentOne software (mean difference = 5.73 ± 0.96 mm and 6.95 ± 1.26 mm, respectively). The mesial translation of the maxillary and mandibular molars in Ortho simulation setup (8.35 ± 1.62 mm and 8.69 ± 1.91 mm, respectively) and dentOne setup (7.41 ± 1.28 mm and 7.74 ± 1.90 mm, respectively) was statistically significantly higher than that obtained using the manual setup (− 0.08 ± 0.27 mm, 0.03 ± 0.47 mm, respectively). All setups showed clinically significant lingual inclination of maxillary and mandibular incisors, with the manual setup exhibiting more lingual inclination than both automated setups. Ortho Simulation setup was the fastest method (4.14 ± 0.53 min), followed by dentOne automated setups (7.57 ± 0.94 min), then the manual setup (21.00 ± 1.66 min).

**Conclusion:**

Despite being faster, the automated diagnostic setups for bimaxillary protrusion cases constricted the dental arch and did not manage the extraction spaces well, hence, simulating anchorage loss. These findings highlight the need for manual refinement of the automated setups.

**Supplementary Information:**

The online version contains supplementary material available at 10.1186/s40510-026-00605-6.

## Background

Orthodontic diagnostic setup is a procedure that was first described by Kesling [1]. The diagnostic setup enables the orthodontist to visualize possible treatment outcomes, predict potential limitations, and choose the best treatment strategy [1]. Hou et al. [2] showed that visualizing diagnostic setups can alter the orthodontic treatment planning decisions and increase the practitioners’ confidence.

Traditionally, the diagnostic setup was made using plaster casts [1]. However, making the plaster models and the diagnostic wax-up entails extra laboratory work and extra time [3]. Digital diagnostic setup was shown to be as reliable and accurate as the manual setup using plaster models in non-extraction and extraction cases [4–6]. Furthermore, the virtual setup makes it easy to discuss the treatment alternatives and expected treatment results with the patients and other dental professionals [7]. Moreover, the same digital model allows for multiple treatment simulations where no actual cutting of the models is needed to allow teeth repositioning since teeth are cut from the model using virtual segmentation, which was previously shown to be accurate [8].

Multiple software packages that offer functions for segmentation of digital dental models, three-dimensional tooth repositioning, and artificial intelligence (AI) based virtual setup are now available. With the development of AI technologies to automate orthodontic procedures that were conventionally done manually, more investigations are needed to evaluate the ability of AI to replace the time-consuming manual steps and to ascertain its applicability in the clinical practice.

Previous research has compared automated digital setup and manual digital setup in non-extraction cases [3]. However, to date, no study compared manual digital setup and automated digital setup in extraction cases. The diagnostic setup in extraction cases entails a decision-making process regarding the anchorage requirements of the case. Additionally, it is important to preserve the patient’s original arch form, inter-canine width (ICW) and inter-molar width (IMW), despite the extractions, to ensure stability of the results post-treatment [9, 10].

Therefore, the aim of the current study was to evaluate automated digital diagnostic setup in bimaxillary dentoalveolar protrusion extraction cases using two different software packages and to compare it to manual digital setup. The null hypothesis assumed that there will be no significant differences between the two approaches regarding dental arch changes, quantity and quality of tooth movement, as well as the time needed to perform the virtual setup.

## Methodology

### Study subjects

This in silico study was conducted in the department of Orthodontics, Faculty of Dentistry, Alexandria University, following ethical approval granted by the faculty’s Institutional Review Board (IORG: 0008839, protocol no. 0730-07/2023). Intraoral scans were used to address the aim of the study. The scans were obtained from patients with the following inclusion criteria: 1- Presence of bimaxillary dentoalveolar protrusion with an orthodontic treatment plan including extraction of the four first premolars with absolute anchorage [11], 2- Presence of a full set of permanent dentition, excluding third molars, 3- Normodivergent cases, and 4- Good oral hygiene. The exclusion criteria were presence of partially erupted teeth, supernumerary teeth, missing teeth (except third molars), dental prosthesis or any syndromes or craniofacial anomalies.

### Sample size calculation

Sample size calculation was performed assuming a 95% confidence level, a 5% alpha error, and a statistical power (1–β) of 80%, to detect differences in (ICW) between manual and virtual setups using a paired-sample design. Im et al. [5] reported mean (95% CI) maxillary ICW = 37.65 mm (36.87, 38.42) for manual setup, and 37.07 mm (36.18, 37.98) for the virtual setup with a mean difference (95% CI) = 0.57 mm (− 0.01, 1.15) and a standard deviation of the difference scores (SD = 0.94). Based on these parameters, the sample size was calculated to be 13 patients, increased to 14 to make up for procedural problems [12]. Sample size estimation was calculated using MedCalc Statistical Software version 19.0.5 (MedCalc Software bvba, Ostend, Belgium).

### Procedures

Full records, including pre-treatment extraoral and intraoral photographs, lateral cephalometric radiographs, panoramic radiographs and full-arch intraoral scans, were taken for the eligible cases. The intraoral scans were obtained using CEREC Omnicam intra-oral scanner and its software Connect SW, version 5.2.7, 2023 (Sirona Dental Systems GmbH, Bensheim, Germany) and exported in Standard Tessellation Language (STL) file format. The STL files were imported into Ortho Simulation software (MEDIT Corp, Seoul, South Korea) and dentOne^®^ software (DIORCO co. ltd, Yongin, South Korea).

All the setup procedures were performed by one researcher (TK) and checked by another experienced orthodontist (YY).

#### Manual DentOne digital setup

**Fig. 1:**
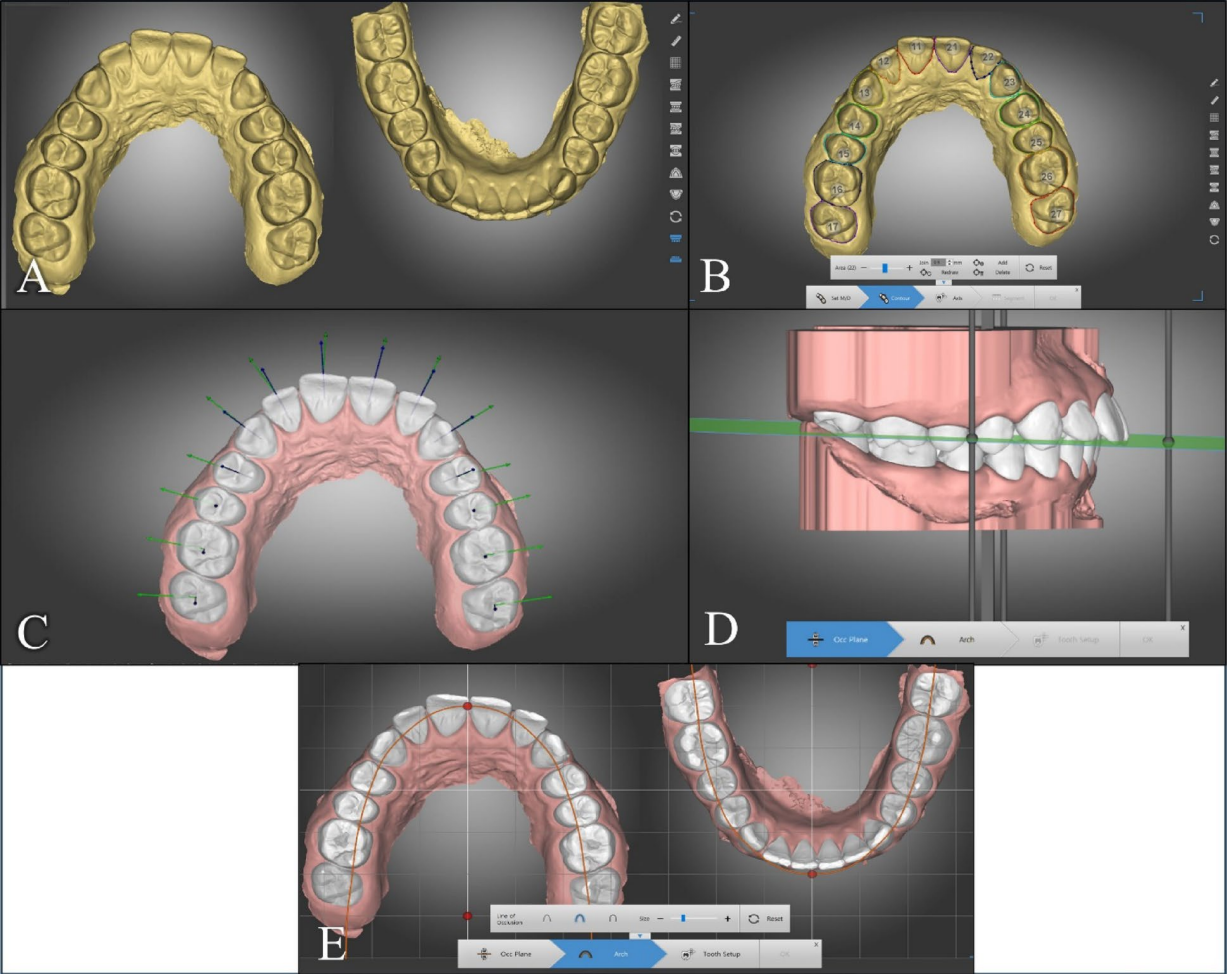
Preparation for the manual diagnostic setup in dentOne^®^ software: **A** Imported scanned data; **B** Teeth segmentation; **C** Checking the axes of the clinical crowns; **D** Setting the occlusion plane; **E** Setting the arch form

**Fig. 2:**
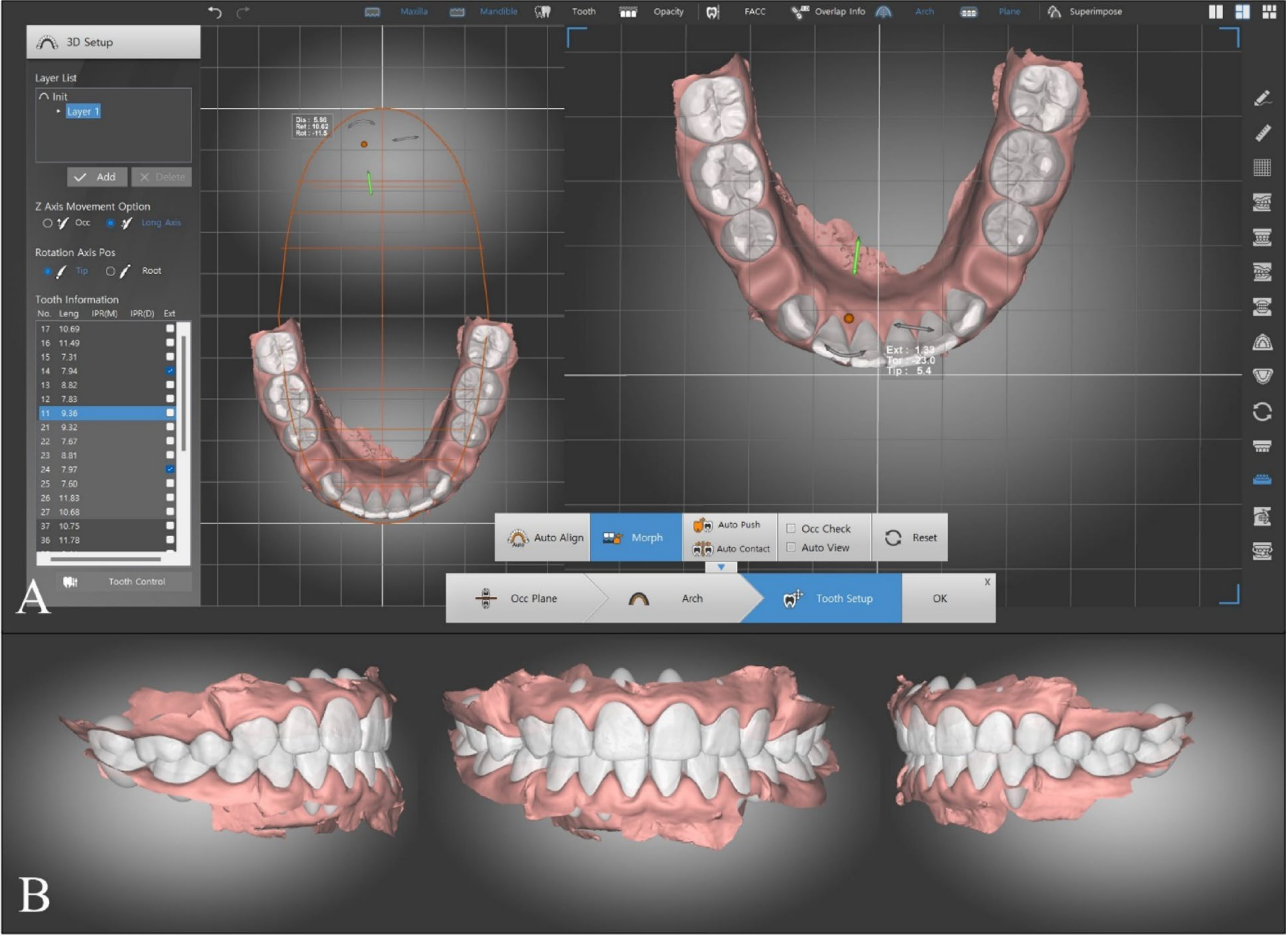
Manual diagnostic setup in dentOne^®^ software: **A** Leveling and alignment utilizing the extraction space; **B** Final setting after space closure by absolute anchorage

A virtual manual setup was conducted for each patient using dentOne^®^ software after importing the intraoral scans as shown in Fig. 1A. Segmentation of the teeth was performed automatically using the software, then adjusted manually if needed (Fig. 1B). The following step was adjustment of the axes of the clinical crowns (Fig. 1C). The occlusal plane of each case was then defined by the researcher (Fig. 1D), and the appropriate arch form was set individually for each case (Fig. 1E). The dental setup was performed manually according to the planned treatment scenario of extracting the four first premolars as shown in Fig. 2A. The teeth were leveled and aligned utilizing the extraction space. Then space closure was performed according to the anchorage requirements and the incisors’ end-of-treatment goal positions (Fig. 2B). The dental setup was checked by another orthodontist with more than 10 years of experience (YY) to ensure it exhibited normal intramaxillary and intermaxillary relations following Andrews’ six keys of normal occlusion [13].

#### Automated DentOne digital setup

**Fig. 3:**
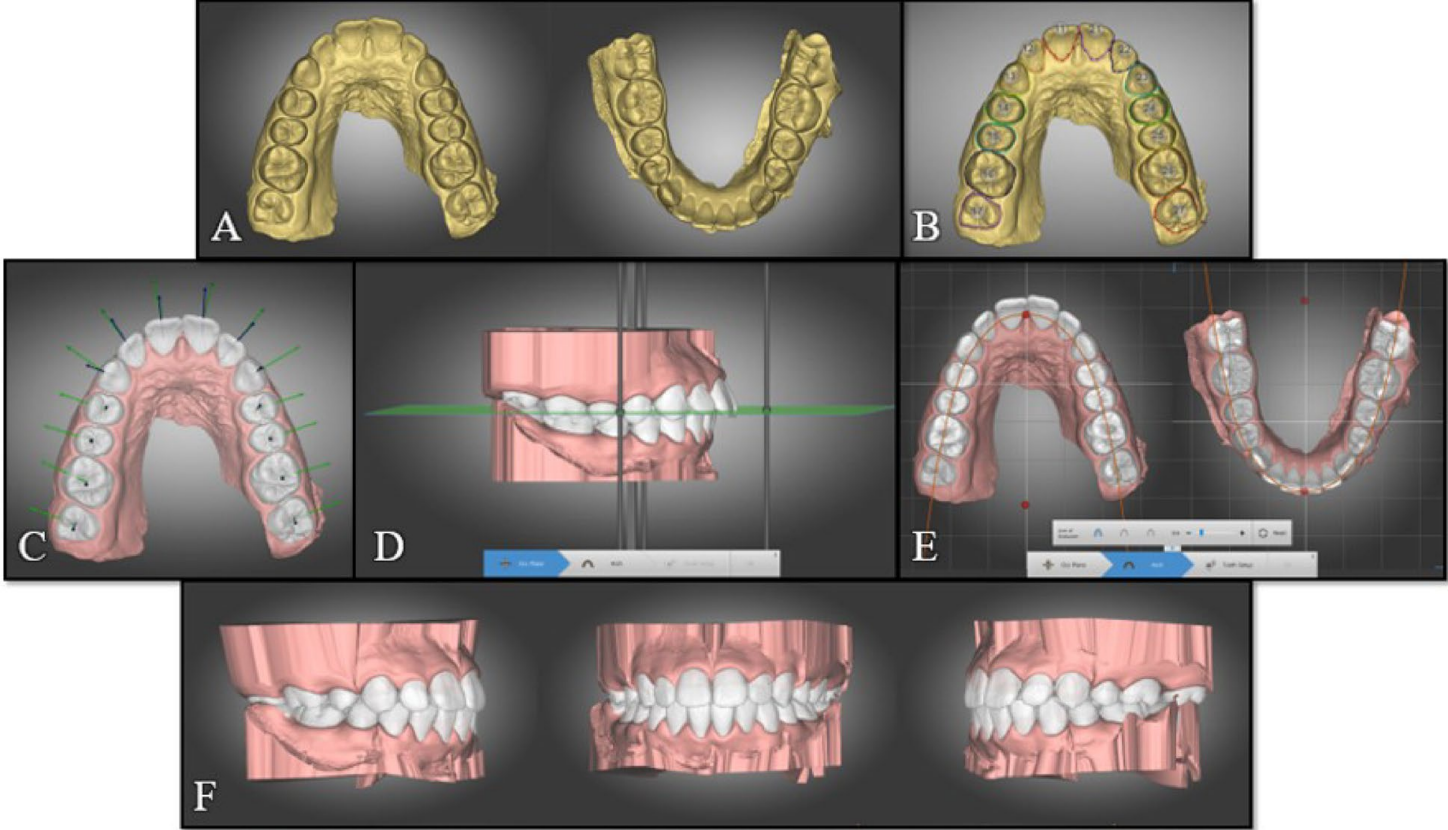
Automatic diagnostic setup in dentOne^®^ software: **A** imported scanned data; **B** Teeth segmentation; **C** Checking the axes of the clinical crowns; **D** Setting the occlusion plane; **E** Setting the arch form. **F** Final automated setup

For the automated digital setups done using dentOne^®^ software, a new patient record was created and the STL files were imported again. The software automatically performed teeth segmentation and detected the facial axes of the clinical crowns. The researcher (TK) adjusted the segmentation and facial axes if needed then set the occlusion plane and the arch form. After identifying the teeth to be extracted, the researcher selected the “Auto align” function and the software performed the alignment and space closure automatically without further intervention from the researcher (Fig. 3).

#### Automated ortho simulation digital setup

**Fig. 4:**
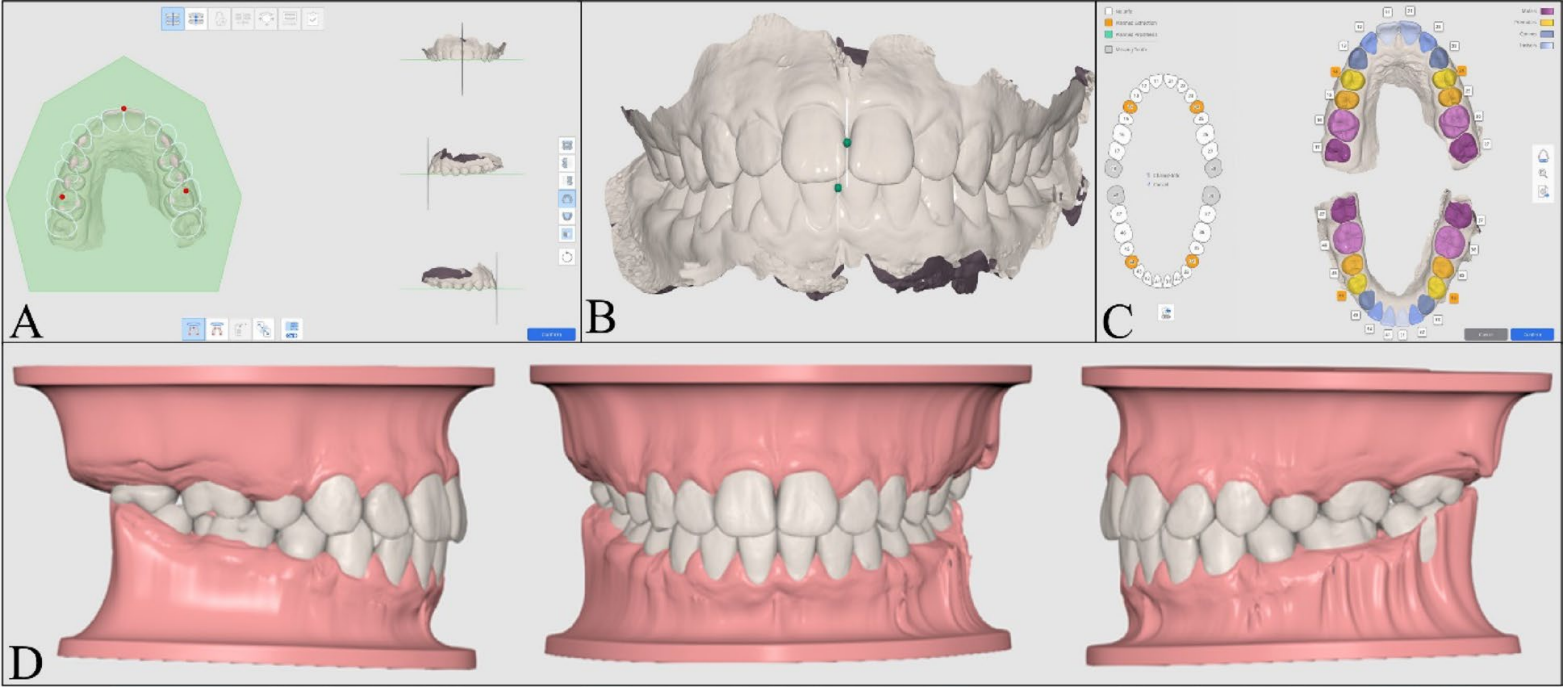
Automatic diagnostic setup in Ortho Simulation software **A** Identification of 3 occlusal reference points on the upper arch to orient the digital model; **B** upper and lower dental midlines are defined by the operator; **C** The software performs automatic teeth segmentation, and the clinician identifies the four first premolars to be extracted during the setup; **D** The software performs automatic virtual setup

For the automated setups done using Ortho Simulation software, after importing the intraoral scans (Fig. 4A), the maxillary and mandibular dental midlines were defined (Fig. 4B) then the dental units were segmented automatically by the software and were adjusted by the researcher if needed (Fig. 4C). The researcher then defined the teeth to be extracted, and the software automatically performed the digital setup (Fig. 4D).

### Outcome measures

**Fig. 5:**
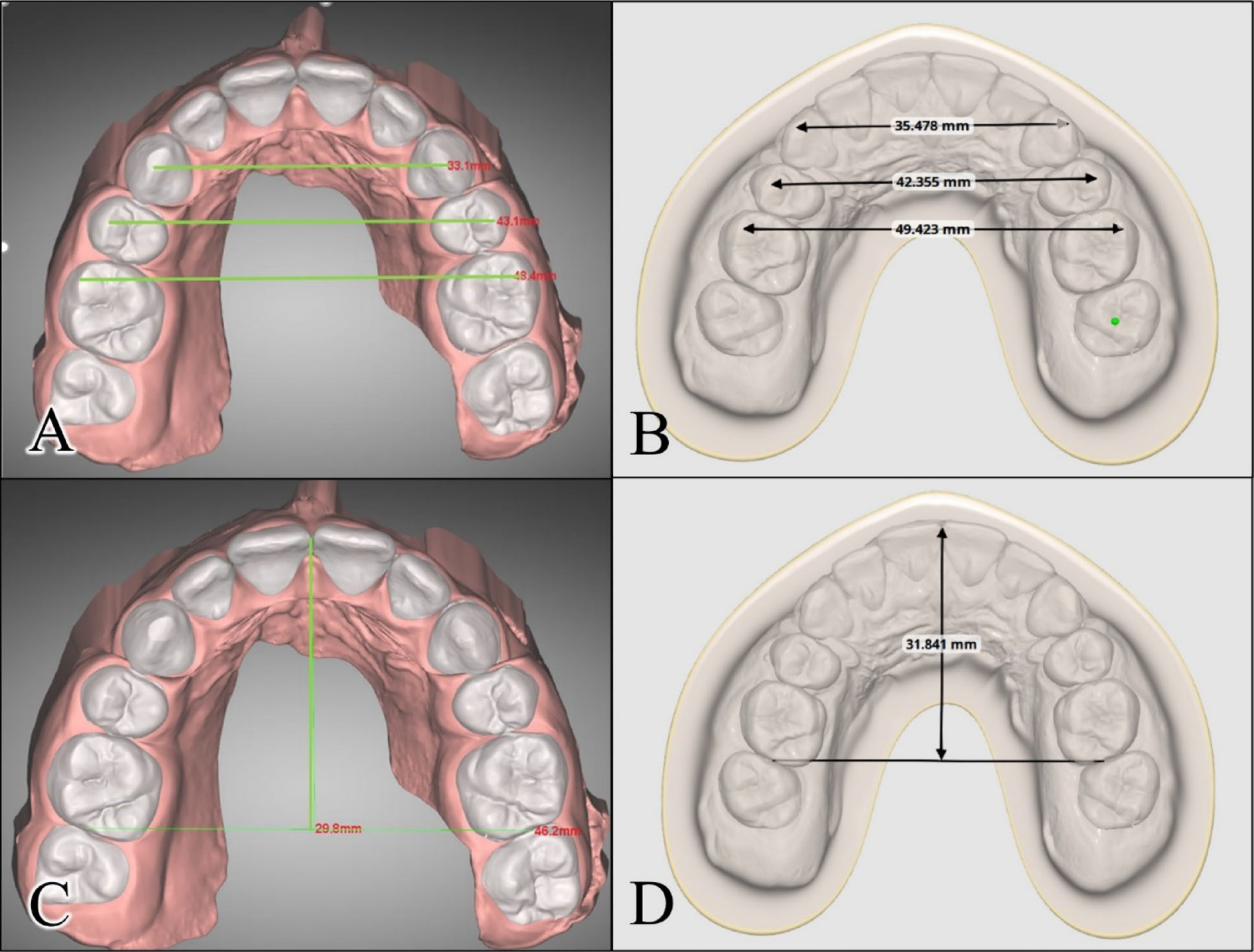
Measurement of the dental arch parameters: Measurement of intercanine, interpremolar, and intermolar widths in **A** DentOne software and **B** Medit software; Measurement of arch length in **C** DentOne software and **D** Medit software

The following parameters were evaluated for each case:

1. Dental arch parameters: The inter-canine width (ICW), inter- premolar width (IPW), inter-molar width (IMW), and arch length (AL) before and after the setup were measured, in mm, using dentOne^®^ software and Medit design software (MEDIT Corp, Seoul, South Korea) as shown in Fig. 5. The definitions of the linear variables are outlined in Table 1.

**Table 1:** The definitions of the measured dental arch parameters

Parameter	Definition
Inter-canine width (ICW)	Measured as the linear distance between the tips of the permanent canines.
Inter-premolar width (IPW)	Measured as the linear distance between the cusp tips of the left and right second premolars.
Inter-molar width (IMW)	Measured as the linear distance between the mesiobuccal cusp tips of the right and left first permanent molars.
Arch length (AL)	Measured as the perpendicular linear distance between an occlusal point midway between the central incisors to a line connecting the midpoints of the distal surfaces of the first permanent molars.
2. Dental movements: The facio-lingual, mesio-distal and occluso-gingival linear movements, in mm, as well as the change in crown rotation, mesio-distal crown tip, and facio-lingual crown torque, in degrees, were reported by each software following the diagnostic setup (Fig. 6) [3]. The values were recorded by the researcher so that positive linear values indicated buccal, mesial, or occlusal movements; while negative values indicated lingual, distal, or gingival movements. For the angular measurements, positive values indicated mesial out rotation, buccal crown torque, and mesial crown tip while the negative values indicated mesial in rotation, lingual crown torque and distal crown tip.

**Fig. 6:**
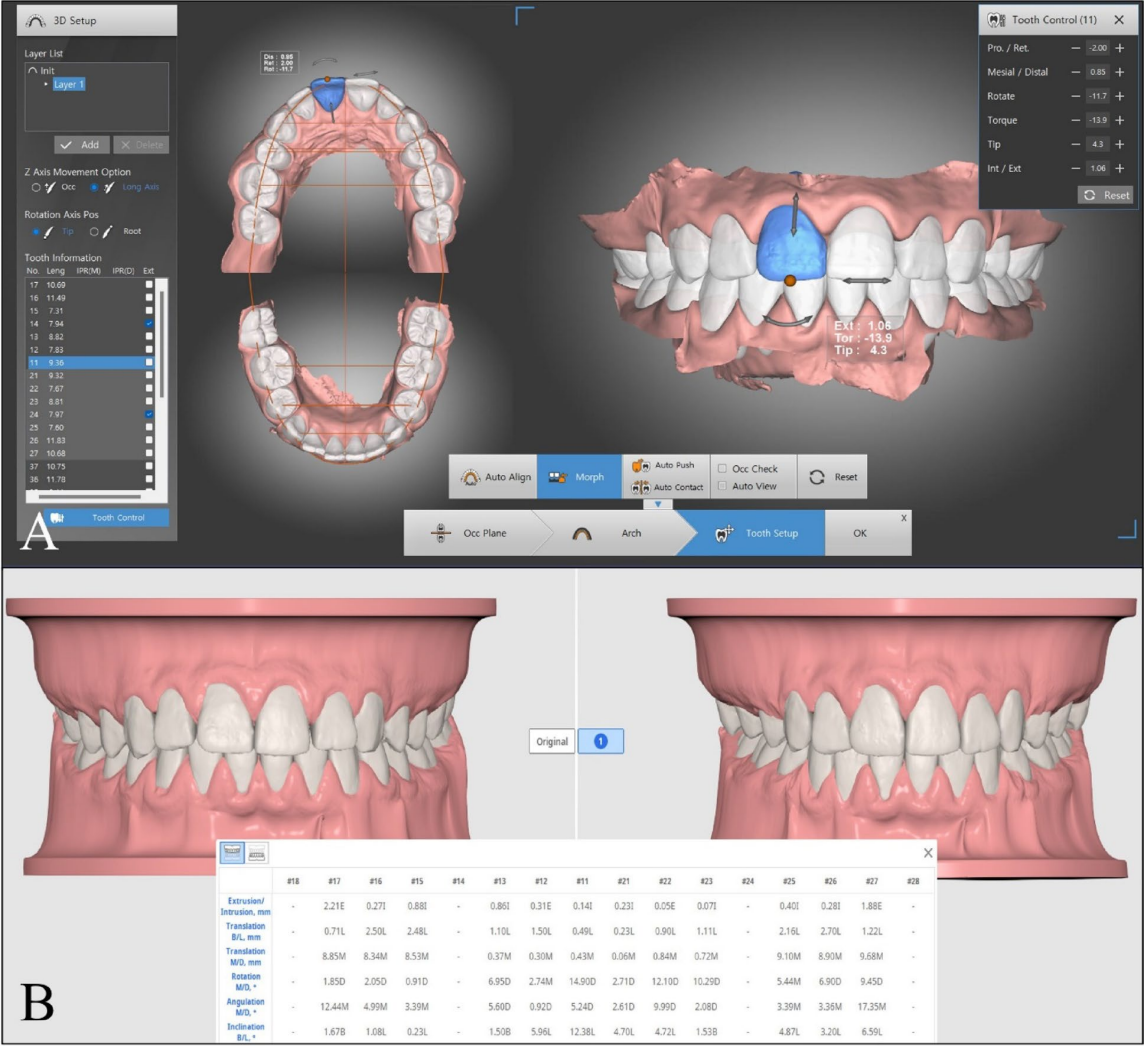
Dental movements in **A** dentOne^®^ software; and **B** Ortho Simulation software
  When interpreting the results, the threshold for clinical significance was set at 0.5 mm for linear displacements and 2° for angular measurements [14].
3. Time needed to perform the digital setup: The time was recorded, in minutes, starting from the moment the intraoral scans were imported into the software and ending when the setup was completed.

### Reliability

Intra-examiner reliability for the measured dental arch parameters was assessed by randomly selecting five STL patient files from each study group and repeating the measurements by the same researcher (TK) on each software. Moreover, inter-examiner reliability was assessed by repeating the measurements on five randomly selected STL files by another researcher (YY). Intraclass Correlation coefficient (ICC) was calculated to determine the reliability of the measurements.

### Statistical analysis

Normality of all parameters was checked using Shapiro Wilk test and Q-Q plots. All data were presented using mean and standard deviation (SD). Friedman test followed by pairwise comparisons with Bonferroni correction to adjust for type I error was used for comparison between the different groups for the linear and angular tooth movements. Differences in arch dimension measurements from baseline values within each software were assessed using paired *t*-test, while the mean differences across the software programs were analyzed using Friedman test.

One Way ANOVA followed by Tukey’s post hoc test with Bonferroni correction were employed for time analysis.

All tests were two tailed and the significance level was set at *p* value < 0.05. Data were analyzed using IBM SPSS, for Windows, version 23, Armonk, NY, USA and MedCalc Statistical Software version 19.2.6 (MedCalc Software bv, Ostend, Belgium; https://www.medcalc.org/en/).

## Results

The overall intraclass correlation coefficient (ICC) ranged between 0.801 and 0.992, reflecting high consistency for intra-examiner reliability. Similarly, inter-examiner reliability showed high agreement between examiners, with ICC ranging between 0.983 and 0.999 [15] (Supplementary table S1).

### Arch dimensions

The changes in arch parameters are shown in Table 2. All the arch dimensions significantly decreased following automated setup using Medit Ortho Simulation. Similarly, all the arch dimensions, except the ICW, significantly decreased following automated setup using dentOne. Conversely, the arch parameters of the manually generated setups did not show statistically significant changes compared to the pre-setup measurements, except for the arch length, which decreased significantly post-setup.

**Table 2:** Comparison of the arch dimensions, in mm, before (baseline) and after automated and manual setups

	Arch parameters	Baseline	Ortho Simulation	dentOne	Manual	Mean difference ± SD			
		Mean ± SD	Mean ± SD	Mean ± SD	Mean ± SD	Ortho Simulation-Baseline	DentOne-Baseline	Manual-Baseline	*P*-value^1^
Upper	ICW	35.30 ± 2.93	34.00 ± 2.16	34.71 ± 1.68	37.75 ± 2.13	− 1.30 ± 1.62	− 0.59 ± 2.42	0.45 ± 1.25	0.003*
	*p* value					0.010*	0.376	0.203	
	IPW	46.99 ± 4.22	40.63 ± 2.65	43.69 ± 1.75	46.46 ± 3.36	− 6.37 ± 2.86	− 3.31 ± 3.12	− 0.53 ± 1.28	< 0.001*
	*p* value					< 0.001*	0.002*	0.146	
	IMW	51.61 ± 4.07	44.90 ± 6.71	47.76 ± 1.58	51.56 ± 3.42	− 6.70 ± 5.82	− 3.85 ± 3.10	− 0.04 ± 1.16	< 0.001*
	*p* value					0.001*	< 0.001*	0.892	
	AL	39.14 ± 2.71	31.21 ± 1.30	30.08 ± 2.06	29.59 ± 2.04	− 7.93 ± 2.35	− 9.06 ± 2.70	− 9.54 ± 2.52	0.017*
	*p* value					< 0.001*	< 0.001*	< 0.001*	
Lower	ICW	27.44 ± 2.15	26.09 ± 1.61	27.15 ± 0.73	27.56 ± 1.77	− 1.35 ± 1.84	− 0.29 ± 1.85	0.12 ± 0.65	0.019*
	*p* value					0.017*	0.564	0.494	
	IPW	39.36 ± 4.48	33.12 ± 2.79	35.36 ± 1.17	39.06 ± 3.23	− 6.24 ± 3.16	− 3.99 ± 3.86	− 0.30 ± 1.90	< 0.001*
	*p* value					< 0.001*	0.002*	0.565	
	IMW	44.56 ± 4.06	38.22 ± 3.06	40.16 ± 1.20	44.65 ± 3.67	− 6.34 ± 2.68	− 4.41 ± 3.34	0.09 ± 0.76	< 0.001*
	*p* value					< 0.001*	< 0.001*	0.678	
	AL	34.61 ± 2.29	27.53 ± 1.02	28.59 ± 1.57	26.33 ± 1.28	− 7.08 ± 1.74	− 6.03 ± 1.50	− 8.29 ± 1.84	< 0.001*
	*p* value					< 0.001*	< 0.001*	< 0.001*	

The bold values in the tables indicate statistically significant results

*Statistically significant difference at *p* value < 0.05

*SD* Standard deviation; *ICW* Inter-canine width; *IPW* Inter-premolar width; *IMW* Inter- molar width; *AL* Arch length

*P*-value: Paired *t*-test, *p* value^1^: Friedman test

The changes in arch parameters following setup were significantly different between the three methods (Table 2). Post hoc comparisons of the reported changes are reported in Supplementary table S2.

### Linear tooth movements

The amount of linear tooth movement following the digital setup and the differences between the three methods are reported in Table 3. Statistically significant differences were found between the three methods for all the linear tooth movements.

**Table 3:** Comparison of linear tooth movements between the automated and manual digital setups

	Tooth type	Linear tooth movement, mm				Mean difference between the groups, mm					
		Ortho simulation	dentOne	Manual	*P*-value	Ortho simulation versus dentOne	*P*-value^1^	Ortho simulation versus manual	*P*-value^1^	dentOne versus manual	*P*-value^1^
		Mean ± SD				Mean ± SD		Mean ± SD		Mean ± SD	
Facio-lingual translation	Upper incisors	− 1.00 ± 0.73	− 1.26 ± 1.61	− 8.25 ± 2.15	< 0.001*	0.24 ± 1.42	1.00	5.97 ± 1.10	< 0.001*	5.73 ± 0.96	< 0.001*
	Upper canines	− 0.95 ± 0.64	− 0.82 ± 1.60	− 3.67 ± 1.12	< 0.001*	− 0.13 ± 1.57	1.00	2.73 ± 1.09	< 0.001*	2.85 ± 0.85	< 0.001*
	Upper premolars	− 1.25 ± 1.02	0.53 ± 1.79	− 0.33 ± 0.70	< 0.001*	− 1.78 ± 1.46	< 0.001*	− 0.92 ± 0.87	0.004*	0.86 ± 1.35	0.326
	Upper molars	− 1.34 ± 1.72	− 0.84 ± 1.82	− 0.34 ± 0.68	< 0.001*	− 0.51 ± 2.18	0.070	− 1.00 ± 1.79	< 0.001*	− 0.49 ± 1.52	0.070
	Lower incisors	1.28 ± 1.14	1.04 ± 1.68	− 7.13 ± 2.10	< 0.001*	0.07 ± 1.23	1.00	7.02 ± 1.29	< 0.001*	6.95 ± 1.26	< 0.001*
	Lower canines	− 0.10 ± 1.45	0.24 ± 0.134	− 4.16 ± 0.98	< 0.001*	− 0.34 ± 1.14	1.00	4.07 ± 1.22	< 0.001*	4.41 ± 1.06	< 0.001*
	Lower premolars	− 1.16 ± 1.36	0.89 ± 2.20	− 0.25 ± 1.49	< 0.001*	− 2.05 ± 1.40	< 0.001*	− 0.91 ± 0.94	0.033*	1.14 ± 1.34	0.003*
	Lower molars	− 1.32 ± 1.12	− 1.03 ± 1.95	− 0.05 ± 0.32	< 0.001*	− 0.29 ± 2.02	1.00	− 1.27 ± 1.16	< 0.001*	− 0.98 ± 1.90	< 0.001*
Mesio-distal translation	Upper incisors	− 0.23 ± 0.54	− 0.48 ± 0.80	− 6.33 ± 1.78	< 0.001*	0.42 ± 1.19	1.00	7.19 ± 1.37	< 0.001*	6.77 ± 1.31	< 0.001*
	Upper canines	− 0.15 ± 1.12	− 0.48 ± 1.18	− 7.81 ± 0.69	< 0.001*	0.33 ± 1.38	1.00	7.67 ± 1.37	< 0.001*	7.34 ± 1.34	< 0.001*
	Upper premolars	7.82 ± 1.44	6.87 ± 3.07	− 0.12 ± 0.30	< 0.001*	0.95 ± 3.18	1.00	7.94 ± 1.48	< 0.001*	6.99 ± 3.03	< 0.001*
	Upper molars	8.35 ± 1.62	7.41 ± 1.28	− 0.08 ± 0.27	< 0.001*	0.94 ± 2.01	0.771	8.43 ± 1.60	< 0.001*	7.49 ± 1.32	< 0.001*
	Lower incisors	0.20 ± 1.14	0.46 ± 1.42	− 6.35 ± 2.48	< 0.001*	− 0.40 ± 0.88	0.176	7.08 ± 2.14	< 0.001*	7.47 ± 2.16	< 0.001*
	Lower canines	0.55 ± 1.56	0.74 ± 1.84	− 8.40 ± 1.15	< 0.001*	− 0.19 ± 1.08	0.855	8.95 ± 1.56	< 0.001*	9.14 ± 1.71	< 0.001*
	Lower premolars	8.42 ± 1.88	8.06 ± 1.94	0.04 ± 0.56	< 0.001*	0.36 ± 1.78	1.00	8.38 ± 1.65	< 0.001*	8.02 ± 1.71	< 0.001*
	Lower molars	8.69 ± 1.91	7.74 ± 1.90	0.03 ± 0.47	< 0.001*	0.95 ± 2.08	0.267	8.66 ± 1.70	< 0.001*	7.71 ± 1.82	< 0.001*
Vertical translation	Upper incisors	0.21 ± 0.62	0.16 ± 1.51	1.12 ± 1.11	< 0.001*	0.17 ± 1.44	0.357	− 0.55 ± 1.18	< 0.001*	− 0.72 ± 1.54	0.048*
	Upper canines	− 0.05 ± 0.93	− 0.26 ± 1.23	0.33 ± 1.20	0.010*	0.21 ± 1.12	1.00	− 0.38 ± 1.52	0.048*	− 0.58 ± 1.67	0.015*
	Upper premolars	0.11 ± 0.82	− 0.68 ± 0.77	− 0.07 ± 0.18	< 0.001*	0.80 ± 0.93	0.001*	0.18 ± 0.89	1.00	− 0.62 ± 0.79	0.002*
	Upper molars	0.72 ± 0.92	0.06 ± 1.60	− 0.1 ± 0.22	< 0.001*	0.66 ± 1.58	0.001*	0.73 ± 0.92	0.002*	0.07 ± 1.56	1.00
	Lower incisors	− 0.99 ± 2.07	− 0.01 ± 2.30	− 0.75 ± 1.46	< 0.001*	− 1.27 ± 1.48	< 0.001*	− 0.57 ± 1.10	0.658	0.70 ± 1.35	0.024*
	Lower canines	− 0.33 ± 1.29	0.39 ± 1.72	− 0.36 ± 1.09	0.031*	− 0.72 ± 1.43	0.184	0.03 ± 1.09	1.00	0.75 ± 1.08	0.033*
	Lower premolars	− 0.56 ± 1.06	0.64 ± 1.13	0.03 ± 0.11	< 0.001*	− 1.20 ± 1.06	< 0.001*	− 0.59 ± 1.04	0.048*	0.61 ± 1.11	0.048*
	Lower molars	− 1.03 ± 0.89	− 0.39 ± 1.89	0.07 ± 0.56	< 0.001*	− 0.65 ± 1.92	0.002*	− 1.10 ± 1.06	< 0.001*	− 0.46 ± 1.92	0.241

*Statistically significant difference at *p* value < 0.05

*SD* Standard deviation

*P*-value: Friedman test, *P*-value^1^: Wilcoxon Signed Rank post-hoc with Bonferroni correction

#### Facio-lingual translation

The manual setup showed lingual translation of the maxillary and mandibular anterior teeth ranging between 3.67 ± 1.12 mm and 8.25 ± 2.15 mm, which was significantly higher than both automated setups (*p* < 0.001).

The automated setup using Ortho simulation resulted in clinically significant lingual movement of the posterior teeth ranging between − 1.16 ± 1.36 mm and − 1.34 ± 1.72 mm which was statistically significant compared to the manual setup. Additionally, the lingual movement of the premolars in Ortho simulation setup was significantly more than that reported for the automated dentOne setup.

#### Mesio-distal translation

**Fig. 7:**
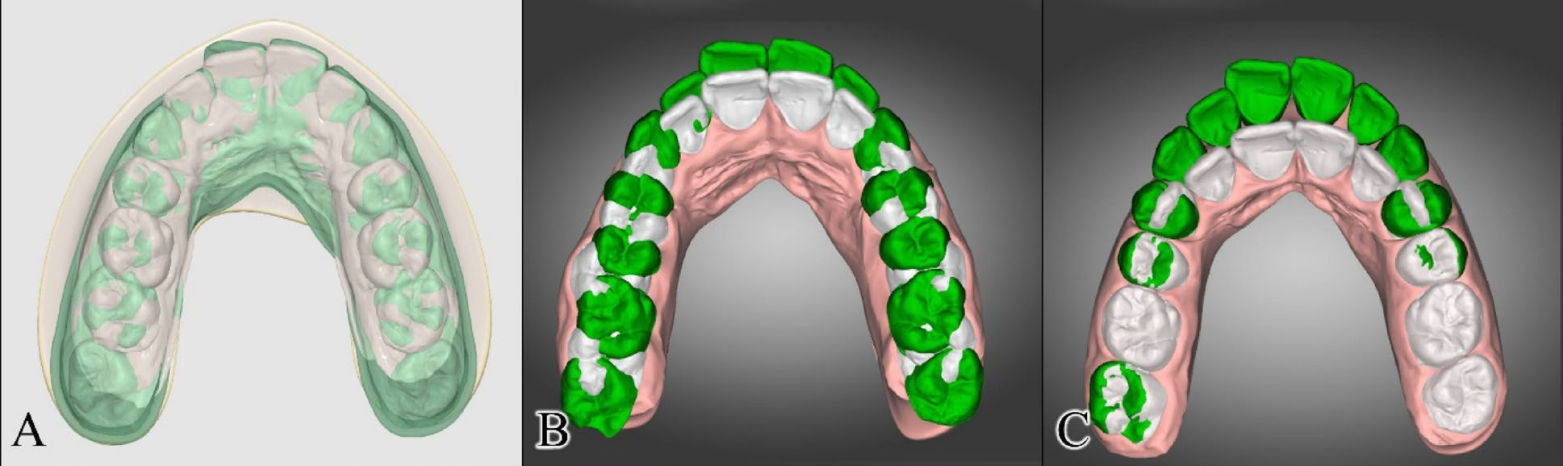
**A** Superimposition of pre-setup (green) and post-setup (white) digital models showing mesial movement of the maxillary posterior teeth following automated setup by Ortho Simulation software; **B** Superimposition of pre-setup (green) and post-setup (white) digital models showing mesial movement of the maxillary posterior teeth following automated setup by dentOne; **C** Superimposition of pre-setup (green) and post-setup (white) digital models showing no mesio-distal translation of the maxillary posterior teeth following manual setup performed using dentOne software

Both automated setups showed clinically significant mesial translation of the maxillary and mandibular posterior teeth ranging between 7.82 ± 1.44 mm and 8.69 ± 1.91 mm in Ortho Simulation models, and between 6.87 ± 3.07 mm and 8.06 ± 1.94 mm in dentOne automated models. Contrarily, in the manual setup, the mesio-distal translation of the posterior teeth ranged between − 0.12 ± 0.30 mm and 0.04 ± 0.56 mm. The mesial translation of the posterior teeth in both automated setups was statistically significantly higher than that obtained using the manual setup (*p* < 0.001). Superimpositions of representative pre-setup and post-setup digital models following automated and manual setup are shown in Fig. 7.

#### Vertical movements

The maxillary anterior teeth were more extruded in the manually executed setup than both automated setups. In the mandibular arch, the lower anterior teeth were more intruded in the manual setup than the automated dentOne setup, but no difference was found in the manual setup compared to Ortho Simulation models.

Clinically negligible vertical movement of the posterior teeth, ranging between − 0.1 ± 0.22 mm and 0.07 ± 0.56 mm, was reported with the manual setup. Ortho Simulation setup exhibited more intrusive movements for the mandibular posterior teeth and more extrusive movements for the maxillary posterior teeth compared to dentOne models.

### Angular movements

Table 4 shows the angular movements reported following the diagnostic setup in each software.

**Table 4:** Comparison of angular movements between automated and manual digital setups

	Tooth type	Angular movements, °				Mean difference between different groups, °					
		Ortho simulation	dentOne	Manual	*P*-value	Ortho simulation versus dentOne	*P*-value^1^	Ortho simulation versus manual	*P*-value^1^	dentOne versus manual	*P*-value^1^
		Mean ± SD				Mean ± SD		Mean ± SD		Mean ± SD	
Rotation	Upper incisors	− 0.85 ± 7.57	1.21 ± 7.17	0.45 ± 7.49	0.495	− 8.02 ± 6.28	–	− 3.41 ± 5.07	–	4.61 ± 6.39	–
	Upper canines	7.14 ± 9.23	− 2.06 ± 8.51	0.01 ± 10.09	< 0.001*	9.20 ± 9.20	< 0.001*	7.12 ± 7.79	0.033*	− 2.07 ± 10.35	0.247
	Upper premolars	− 2.28 ± 5.64	− 5.34 ± 5.69	− 2.79 ± 4.83	0.066	3.06 ± 7.78	–	0.51 ± 3.87	–	− 2.55 ± 7.53	–
	Upper molars	10.08 ± 7.23	0.57 ± 5.75	2.48 ± 3.90	< 0.001*	9.51 ± 6.89	< 0.001*	7.60 ± 6.85	< 0.001*	− 1.91 ± 5.48	0.070
	Lower incisors	− 1.95 ± 8.55	0.33 ± 7.68	1.95 ± 6.48	0.003*	− 4.63 ± 8.02	0.142	− 4.66 ± 7.45	0.002*	− 0.3 ± 6.48	0.469
	Lower canines	1.05 ± 9.34	− 7.28 ± 6.45	− 1.75 ± 8.26	0.001*	8.33 ± 7.34	0.002*	2.80 ± 8.09	1.00	− 5.52 ± 6.65	0.010*
	Lower premolars	6.55 ± 9.81	− 13.91 ± 11.38	2.69 ± 7.53	< 0.001*	20.46 ± 10.40	< 0.001*	3.86 ± 6.99	0.544	− 16.60 ± 11.06	< 0.001*
	Lower molars	4.57 ± 5.53	0.74 ± 4.32	1.73 ± 3.72	< 0.001*	3.83 ± 5.30	< 0.001*	2.84 ± 5.00	< 0.001*	− 0.98 ± 4.75	0.267
Angulation	Upper incisors	− 3.31 ± 6.82	0.40 ± 5.24	− 2.50 ± 6.06	0.015*	− 4.03 ± 7.59	0.062	− 0.46 ± 7.13	1.00	3.57 ± 4.46	0.024*
	Upper canines	− 0.84 ± 4.53	0.36 ± 4.16	− 0.99 ± 5.11	0.134	− 1.19 ± 5.30	–	0.15 ± 5.85	–	1.34 ± 4.03	–
	Upper premolars	− 1.39 ± 4.58	− 0.76 ± 2.55	− 1.01 ± 2.71	0.501	− 0.63 ± 4.59	–	− 0.38 ± 5.05	–	0.25 ± 3.43	–
	Upper molars	4.71 ± 8.36	3.15 ± 4.00	− 0.26 ± 1.72	< 0.001*	1.56 ± 6.32	1.00	4.96 ± 8.08	< 0.001*	3.40 ± 3.88	0.001*
	Lower incisors	0.58 ± 4.88	1.66 ± 3.55	− 0.43 ± 4.03	0.017*	− 2.37 ± 5.30	0.771	1.79 ± 5.27	0.267	4.16 ± 4.95	0.014*
	Lower canines	− 3.02 ± 6.95	5.30 ± 5.12	− 1.14 ± 3.57	< 0.001*	− 8.32 ± 7.39	< 0.001*	− 1.88 ± 7.02	1.00	6.44 ± 5.96	< 0.001*
	Lower premolars	3.33 ± 5.98	1.77 ± 3.46	− 0.19 ± 1.10	0.002*	1.56 ± 6.01	1.00	3.52 ± 5.74	0.002*	1.96 ± 3.36	0.033*
	Lower molars	− 5.17 ± 6.31	− 2.31 ± 3.97	− 0.03 ± 1.04	< 0.001*	− 2.85 ± 4.35	0.018*	− 5.13 ± 6.36	< 0.001*	− 2.28 ± 4.09	0.042*
Inclination	Upper incisors	− 5.37 ± 5.88	− 9.10 ± 4.94	− 16.22 ± 6.56	< 0.001*	5.41 ± 4.48	< 0.001*	12.95 ± 5.35	< 0.001*	7.53 ± 4.37	< 0.001*
	Upper canines	− 1.25 ± 5.48	− 6.54 ± 5.22	− 10.86 ± 6.11	< 0.001*	5.29 ± 4.29	< 0.001*	9.61 ± 7.76	< 0.001*	4.32 ± 7.83	1.00
	Upper premolars	− 3.82 ± 4.53	3.57 ± 6.61	0.20 ± 0.52	< 0.001*	− 7.39 ± 4.45	< 0.001*	− 4.02 ± 4.51	< 0.001*	3.37 ± 6.58	0.326
	Upper molars	− 4.74 ± 5.23	− 7.11 ± 11.84	− 0.20 ± 1.40	< 0.001*	2.37 ± 11.68	1.00	− 4.54 ± 5.14	0.005*	− 6.91 ± 12.03	< 0.001*
	Lower incisors	− 9.77 ± 7.73	− 11.61 ± 8.53	− 15.34 ± 5.26	< 0.001*	2.40 ± 4.38	0.357	8.57 ± 6.53	< 0.001*	6.17 ± 7.74	0.002*
	Lower canines	− 7.73 ± 8.24	− 10.84 ± 8.19	− 13.96 ± 5.38	0.009*	3.11 ± 5.20	0.069	6.23 ± 8.72	0.010*	3.12 ± 9.11	1.00
	Lower premolars	− 4.62 ± 5.65	− 0.89 ± 6.76	1.07 ± 3.82	< 0.001*	− 3.73 ± 7.23	0.018*	− 5.69 ± 5.55	< 0.001*	− 1.96 ± 5.41	0.544
	Lower molars	1.02 ± 5.81	− 5.71 ± 14.85	− 0.02 ± 0.58	0.640	6.73 ± 15.90	–	1.04 ± 5.89	–	− 5.69 ± 14.76	–

*Statistically significant difference at *p* value < 0.05

*SD* Standard deviation

*P*-value: Friedman test, *P*-value^1^: Wilcoxon Signed Rank post-hoc Bonferroni correction

#### Rotational movements

The dentOne automated setups, showed significantly more mesial-in rotation of the mandibular canines and premolars than the manually performed setups (mean difference= − 5.52 ± 6.65° and − 16.60 ± 11.06° respectively).

In Ortho Simulation models, the maxillary and mandibular molars and the maxillary canines showed significantly more mesial out rotation than the manually performed setups (mean difference = 7.60 ± 6.85°, 2.84 ± 5.00°, 7.12 ± 7.79°, respectively), whereas the mandibular incisors showed mesial in rotation when compared to the manual models (mean difference= − 4.66 ± 7.45°).

Comparison of the two automated setups demonstrated significantly more mesial out rotation of the maxillary canines and molars, and the mandibular canines, premolars and molars in Ortho Simulation software compared to dentOne software.

#### Mesio-distal angulation

The automated setup using both software resulted in significantly more mesial tipping of the maxillary molars and mandibular premolars than the manual setup. In contrast, the mandibular molars showed significantly more distal tipping in the automated setups than the manual setup.

Comparison of the automated and manual setups using dentOne software showed more significant distal tipping of the maxillary incisors following the manual setup, and more significant mesial tipping of the mandibular incisors following the automated setup.

#### Facio-lingual inclination

The maxillary and mandibular anterior teeth showed clinically significant lingual inclination in all the setups. More significant lingual inclination of the incisors was reported in the manual setup than both automated approaches. Additionally, significantly more lingual inclination of the maxillary incisors and canines was reported in dentOne automated setups than in Ortho Simulation setup.

The change in inclination of the posterior teeth in the manual setup was negligible ranging between (− 0.20 ± 1.40° and 1.07 ± 3.82°). In contrast, notable positive and negative changes in the inclination of the posterior teeth were observed in both automated setups.

### Duration

The duration required to perform the setup differed significantly between the three methods as shown in Table 5. The manual setup required the longest time (21.00 ± 1.66 min), followed by automated setups by dentOne (7.57 ± 0.94 min). The fastest setup was performed using Ortho Simulation software (4.14 ± 0.53 min).

**Table 5:** Comparison of time duration to perform the setup in the three groups

Duration, min				Mean difference ± SD					
Ortho simulation	dentOne	Manual	*P* value	Ortho simulation - dentOne	*P*-value^1^	Ortho simulation -manual	*P*-value^1^	dentOne-manual	*P*-value^1^
Mean ± SD				Mean ± SD		Mean ± SD		Mean ± SD	
4.14 ± 0.53	7.57 ± 0.94	21.00 ± 1.66	< 0.001*	− 3.43 ± 0.76	< 0.001*	− 16.86 ± 1.61	< 0.001*	− 13.43 ± 1.60	< 0.001*

*Statistically significant difference at *p* value < 0.05

*SD* Standard deviation

*P* value: One Way ANOVA, *P*-value^1^: Tukey’s post hoc test with Bonferroni correction

##  Discussion

Recently, there has been a growing interest around the world in using artificial intelligence (AI) and digital solutions that promise to save time, decrease human errors and enhance treatment outcomes in the field of orthodontics [16]. AI-based tools, such as software used for analyzing radiographs and for predicting treatment outcomes, are being developed to make diagnosis and treatment planning faster and more personalized [17, 18]. As the use of AI-based technologies is becoming more common in orthodontics, it is important to investigate their capabilities, limitations and clinical implications. Hence, the aim of the study was to evaluate AI-generated diagnostic setups in comparison to a manual digital setup in bimaxillary dentoalveolar protrusion cases to be treated with extractions. Statistically significant differences were found between the automated setups and the manual setups leading to the rejection of the null hypothesis.

Maintaining the ICW and IMW is a key to post-treatment stability [19]. In the present study, the manual setups preserved the patients’ initial inter-canine and inter-molar distances. In contrast, both automated setups resulted in narrower arch forms, particularly in the molar region, where significant lingual translation and inclination were observed. Similarly, Woo et al. [3] reported narrowing of the arch form following an automated setup using Ortho Simulation software in non-extraction cases owing to the lingual inclination of the teeth. The reduction in IMW in the automated setups in the current study may also be a consequence of the molars shifting mesially into a narrower part of the arch after the loss of one dental unit in each quadrant [20]. Previous research has shown that undesired widening or narrowing of the arch, especially at the canines and molars, provoked post-treatment instability [21]. De La Cruz et al. [22] demonstrated that the arch form tends to return towards its pre-treatment shape after retention. They highlighted that the greater the changes in arch form that occur during treatment, the greater the tendency for post-retention relapse. Moreover, narrowing of the dental arch may result in wider buccal corridors, hence, adversely affecting the smile esthetics [23].

In the current study, the arch length significantly decreased in both the manual and the automated setups. The observed reduction in arch length aligns with previous research that reported significant shortening of the dental arch following extraction orthodontic treatment [20, 24, 25]. This is attributed to the removal of a dental unit in each quadrant, as well as to alterations in the incisors’ inclination [20, 24, 25]. A considerably larger decrease in arch length was noted following the manual setup than the automated setups. This may possibly be related to the significantly larger decrease in the bucco-lingual inclination of the incisors following the manual setup.

In the manual setup, the maxillary and mandibular anterior teeth were moved lingually and their bucco-lingual inclination was reduced. The incisors positioning and bucco-lingual inclination in the manual setup was based on findings from the lateral cephalometric radiograph of each patient and on the treatment plan addressing the bimaxillary dentoalveolar protrusion. The aim of orthodontic treatment in such cases is to retract and retrocline the anterior teeth to reduce the facial convexity and lip prominence [26–28]. To the contrary, the automated setups showed buccal translation of the lower incisors. Moreover, the torque reduction of the maxillary and mandibular incisors was less than the reduction obtained using the manual setup. The difference in the bucco-lingual position and inclination of the incisors may be explained by the limited data input to the software. The automated setup was only based on the patients’ occlusion represented by the STL files. The lack of patients’ cephalometric and soft tissue data may have resulted in less-than-optimal results following the automated setups. Nevertheless, a larger reduction in the bucco-lingual inclination of the incisors was observed with the automated dentOne setup than the automated Ortho Simulation setup. The manual fine-tuning of the facial axis in dentOne software may have enabled the software to better adjust the tooth inclination during the setup. This, however, contradicts the results obtained by Woo et al. [3]. In their study, despite adjustment of the crown long-axis in Autolign software (Diorco Co. ltd, Yongin, South Korea) prior to performing the automated setup, more errors were reported in the bucco-lingual position and inclination of the maxillary incisors compared to Ortho Simulation software. The inconsistency between the two studies may have arisen from the difference in the treatment plans simulated by the software, where non-extraction treatment was performed in their study versus extraction treatment in the current study.

**Fig. 8:**
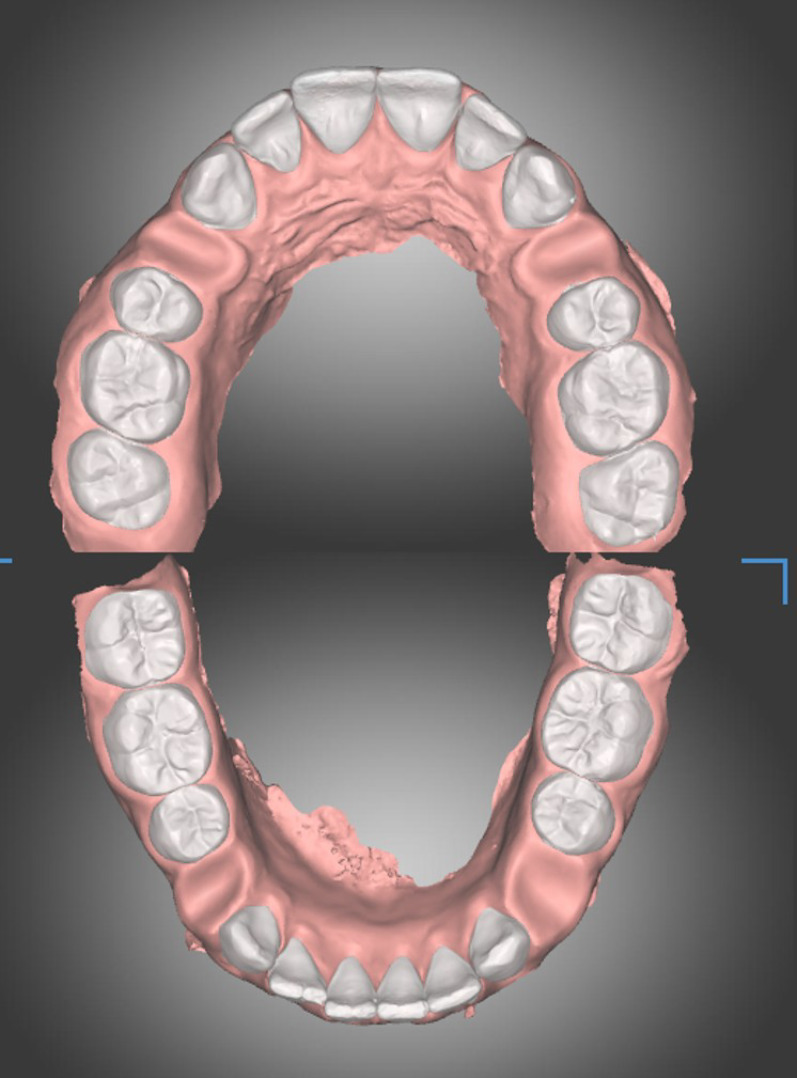
Automated dentOne setup, with the “Template (Molar)” option selected, showing unclosed extraction spaces

In contrast to the manual setup, where the extraction spaces were closed by retraction of the anterior teeth, the extraction spaces in the automated setups were mainly closed by mesial translation of the posterior teeth. The treatment objectives of the cases included in the current study entailed absolute anchorage following extraction of the four first premolars [11, 29, 30]. Hence, when performing the manual setup, the orthodontist avoided mesial movement of the posterior teeth. On the other hand, the anchorage requirements were not accounted for by the automated setups. It is worth noting that an attempt was initially made to prevent the mesial movement of the posterior anchor teeth during the automated setups by choosing the “Template (Molar)” option in dentOne software. However, when this setting was applied, the simulation did not achieve space closure by distalization of anterior teeth. Instead, the anterior segment was only aligned within the arch, leaving the extraction space open as shown in Fig. 8.

All the scans included in the current study were of normodivergent patients, therefore, alterations in the vertical positions of the posterior teeth were generally avoided during manual setups. On the other hand, Ortho Simulation automated setup resulted in clinically significant alterations in the vertical positions of the majority of the posterior teeth. Woo et al. [3] reported similar findings in non-extraction cases using the same software, where the molars were extruded following the automated setup. Anteriorly, a significantly larger amount of extrusion of the maxillary incisors was noted following the manual setup, while the lower incisors were intruded. The manual setup of the incisors was largely based on the individual esthetic requirements of the patients as seen in their extra-oral smiling photographs [31]. Another reason for the observed extrusion of the maxillary incisors is the significant torque reduction that took place during their retraction [31].

More mesial in rotation of the canines and premolars was observed in dentOne automated setup than dentOne manual setup, despite using the same software and employing the same pre-setup procedures, which reflects the disparity between automatic and manual alignment. The standard used for the “Auto align” function in dentOne software is Andrews’ six keys [32], the same principle used when performing the manual setup. However, when performing the manual setup, the clinician made individualized adjustments based on the tooth morphology, the arch form, as well as the inter-proximal and occlusal contacts. The variability in the size and shape of teeth affects their optimal alignment and occlusion [33]. On the same note, the majority of the posterior teeth in Ortho Simulation software exhibited clinically notable mesial out rotation. The incorrect alignment may be related to difficulties in automatically determining the mesiodistal axes of posterior teeth when viewed occlusally [34].

Comparison of the automated and manual setups showed significant differences in the direction and degree of tipping. This result may be attributed to the lack of root information, with the software estimating the long axis of the tooth based solely on the crown morphology. Previous research has shown that the variability in crown morphology hinders consistent automatic determination of the ideal tooth axis [34]. In the same vein, two previous studies tested the accuracy of digital software regarding detection of tooth inclination and found that it was not accurate unless correlated with radiographs [35, 36].

The overall time required for the setup was shorter when using automated methods, with Ortho Simulation software requiring less time than dentOne. This difference is presumably due to the mandatory additional steps involved in generation of dentOne automated models, including detection of the mesiodistal axes and the facial axes of the clinical crowns, as well as setting the occlusal plane and the arch form. A similar finding was reported by Woo et al. [3] who found that Autolign, which followed the same steps as dentOne, required more time to generate the automated models than Ortho Simulation in non-extraction cases.

Notably, digital orthodontic setups are used for visual communication with patients. Ortho Simulation software was particularly designed for consultation purposes [8]. This underscores the importance of how the software showcases the simulation to the patient. The patient’s esthetic expectations may not be fully met if the extraction spaces in the simulation are mainly closed by mesialization of the posterior teeth without adequate retraction of the incisors, and with narrowing of the dental arch [37, 38]. This may negatively impact the patients’ perception of the proposed treatment plan. Therefore, while the software offers quick simulations, the clinician’s oversight is essential to ensure that the simulations are both biomechanically and esthetically reasonable. Hence, manual adjustment by the clinician is an integral part of the digital workflow to ensure accurate and personalized diagnostic digital setups. Additionally, the software could be enhanced by incorporating an option to select the type of anchorage required when performing an automated setup. Such feature would allow the simulation to better reflect the treatment goals.

### Generalizability

The results of the current study only apply to the tested software. Software developers continue to upgrade their products in order to improve their performance and provide additional features. Hence, future research should investigate whether such updates would generate better results.

### Limitations

One limitation of the current study is the lack of root integration. Park et al. [39] showed that relying solely on crown data in virtual simulations can result in inaccuracies in treatment planning. When performing the manual setup, the researcher in the current study relied on root information provided by the panoramic and lateral cephalometric radiographs, nevertheless, it is important to note that variations in the clinical experience of orthodontists may influence the manual setup decisions. Some software platforms now allow superimposition of the STL files of the patient’s intraoral scan onto cone beam computed tomography (CBCT) images to improve the accuracy of root position [40–42]. The impact of the integration of CBCT data with intraoral scans when performing automated and manual virtual setups should be evaluated in future research. Nonetheless, the use of CBCT in orthodontic treatment should be individually justified by assessing whether the expected clinical benefits outweigh its potential risks [43].

Another limitation is that direct comparison of the current study findings with the published literature was challenging owing to the scarcity of studies investigating the performance of commercially available software in automated virtual setups. Moreover, the automated setup algorithm used by the software is not disclosed by the software developers, thus limiting the generalizability of the study results [8]. Finally, the values of tooth movement reported by each software in the current study may not be comparable. Previous research has shown that the amount of tooth movement reported by four different software was significantly different despite carrying out the same virtual setup in all of them [42].

## Conclusions

In the diagnostic setup for bimaxillary dento-alveolar protrusion cases, AI and manual approaches produced different results. The automated diagnostic setups, despite being faster, constricted the dental arch and did not manage the extraction spaces well, hence, simulating anchorage loss. These findings highlight the need for manual refinement of the automated setup to ensure its predictability.

## Supplementary Information

Below is the link to the electronic supplementary material.

Supplementary Material 1.

Supplementary Material 2.

## Data Availability

The datasets used and/or analyzed during the current study are available from the corresponding author upon reasonable request.

## Abbreviations

AI: Artificial intelligence
AL: Arch length
CBCT: Cone beam computed tomography
ICC: Intraclass correlation coefficient
ICW: Inter-canine width
IMW: Inter-molar width
IPW: Inter- premolar width
SD: Standard deviation
STL: Standard Tessellation Language
