# Supplementary material for: Comparison between automated and manual digital diagnostic setups of orthodontic extraction cases: an in silico study
Source: Prog Orthod. 2026 Feb 2;27:2. doi: 10.1186/s40510-026-00605-6 (PMC12864649; doi:10.1186/s40510-026-00605-6)
Supplement: Supplementary file 1 — Supplementary Material 1. [file 40510_2026_605_MOESM1_ESM.docx]

|  | **Manual** | **Automated dentOne** | **Ortho Simulation** | **Overall** |
| --- | --- | --- | --- | --- |
|  | **ICC (95% CI)** | | | |
| Intra-examiner reliability | | | | |
| **ICW** | 0.988 (0.915, 0.999) | 0.944 (0.381, 0.996) | 0.971 (0.551, 0.998) | 0.979 (0.914, 0.995) |
| **IPW** | 0.999 (0.990, 1.00) | 0.990 (0.857, 0.999) | 0.995 (0.923, 1.000) | 0.992 (0.966, 0.998) |
| **IMW** | 0.979 (0.674, 0.999) | 0.959 (0.508, 0.997) | 0.989 (0.833, 0.999) | 0.976 (0.905, 0.994) |
| **AL** | 0.943 (0.122, 0.996) | 0.892 (0.650, 0.993) | 0.723 (0.621, 0.965) | 0.801 (0.698, 0.951) |
| Inter-examiner reliability | | | | |
| **ICW** | 0.998 (0.976, 0.999) | 0.995 (0.990, 0.996) | 0.998 (0.971, 0.999) | 0.998 (0.991, 0.999) |
| **IPW** | 0.997 (0.996, 0.998) | 0.999 (0.977, 0.999) | 0.999 (0.990, 0.999) | 0.999 (0.997, 0.999) |
| **IMW** | 0.998 (0.991, 0.999) | 0.997 (0.901, 0.984) | 0.996 (0.946, 0.999) | 0.997 (0.989, 0.999) |
| **AL** | 0.969 (0.715, 0.998) | 0.989 (0.978, 0.999) | 0.991 (0.866, 0.999) | 0.983 (0.938, 0.995) |

Supplementary table S1: Intra-examiner and inter-examiner reliability for dental arch parameters.

AL: Arch length, CI: Confidence Interval, ICC: Intraclass Correlation Coefficient, ICW: Inter-canine width, IMW: Iner-molar width, IPW: Inter-premolar width.
