# Supplementary material for: Comparison between automated and manual digital diagnostic setups of orthodontic extraction cases: an in silico study
Source: Prog Orthod. 2026 Feb 2;27:2. doi: 10.1186/s40510-026-00605-6 (PMC12864649; doi:10.1186/s40510-026-00605-6)
Supplement: Supplementary file 2 — Supplementary Material 2. [file 40510_2026_605_MOESM2_ESM.docx]

**Supplementary Table S2**: Post hoc comparison of the changes in arch dimensions following the automated and manual setups.

| Arch | Teeth | Change in Ortho Simulation vs Change in dentOne | | Change in Ortho Simulation vs Change in Manual | | Change in dentOne vs Change in Manual | |
| --- | --- | --- | --- | --- | --- | --- | --- |
|  |  | Mean ±SD | *p* value | Mean ±SD | *p* value | Mean ±SD | *p* value |
| Upper | ICW | -0.70 ±1.60 | 0.658 | -1.75 ±1.16 | **0.003*** | -1.04 ±1.68 | 0.113 |
|  | IPW | -3.06 ±1.97 | 0.113 | -5.84 ±2.13 | **<0.001*** | -2.78 ±2.32 | **0.024*** |
|  | IMW | -2.85 ±5.81 | 1.00 | -6.66 ±5.69 | **<0.001*** | -3.81 ±2.54 | **0.007*** |
|  | AL | 1.13 ±1.55 | 0.267 | 1.61 ±1.44 | **0.014*** | 0.49 ±2.33 | 0.771 |
| Lower | ICW | -1.06 ±1.25 | 0.218 | -1.47 ±1.43 | **0.018*** | -0.41 ±1.43 | 1.00 |
|  | IPW | -2.24 ±2.26 | 0.113 | -5.94 ±2.22 | **<0.001*** | -3.69 ±2.69 | **0.024*** |
|  | IMW | -1.94 ±2.46 | 0.558 | -6.43 ±2.66 | **<0.001*** | -4.49 ±3.03 | **0.007*** |
|  | AL | -1.05 ±1.27 | 0.267 | 1.20 ±1.15 | 0.070 | 2.26 ±1.17 | **<0.001*** |

AL, Arch length; ICW, Inter-canine width; IMW, inter-molar width; IPW, inter-premolar width; SD*,* Standard deviation.

Post hoc Wilcoxan signed-rank test was used

*Statistically significant difference at p value<0.05
